# Supplementary material for: Ultrastructural and Immunohistochemical Characterization of Maternal Myofibroblasts in the Bovine Placenta around Parturition
Source: Vet Sci. 2023 Jan 7;10(1):44. doi: 10.3390/vetsci10010044 (PMC9863730; doi:10.3390/vetsci10010044)
Supplement: Supplementary file 1 [file vetsci-10-00044-s001.zip › vetsci-2103594-supplementary.pdf]

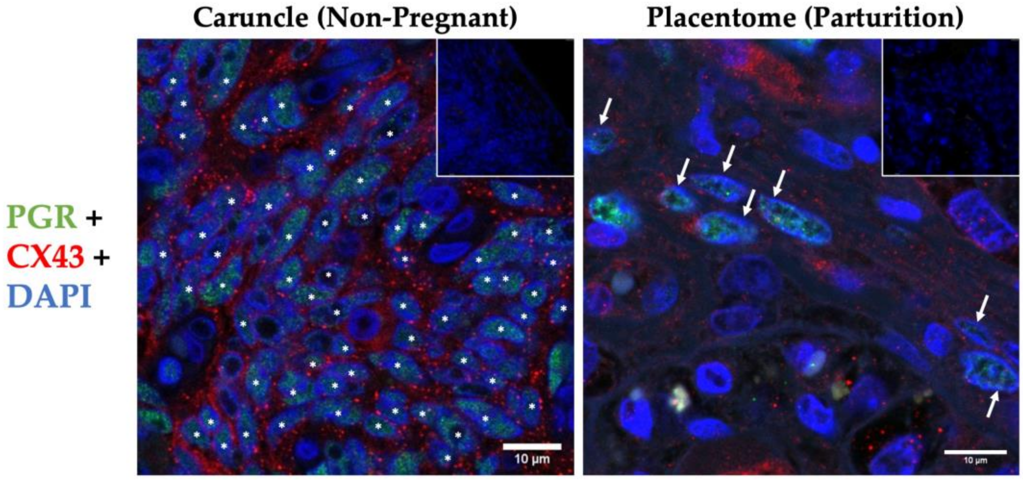

Figure S1. Three-color immunofluorescence staining of maternal stromal cells in caruncular and placentomal tissue sections, stained with DAPI and antibodies against PGR and CX43. Tissue samples of NP caruncles (left figure) or P placentomes (right figure) were stained with PGR (green) and gap junctional marker CX43 (red). In the caruncle, myofibroblasts showed nuclear PGR staining (asterisks) and membrane bound CX43 expression (red dots). In the placentomal stroma, PGR-positive cells (arrows) showed decreased CX43 expression in their membrane borders compared to the caruncle section. Nuclei were counterstained with DAPI (blue). In merged images nuclear signals for PGR and DAPI overlap and appear in brighter white/turquoise color. Weak autofluroescent signal was observed in erythrocytes of surrounding vessels. For each tissue section there was no background staining in the isotype control (insets). Scale bars = 10 µm.

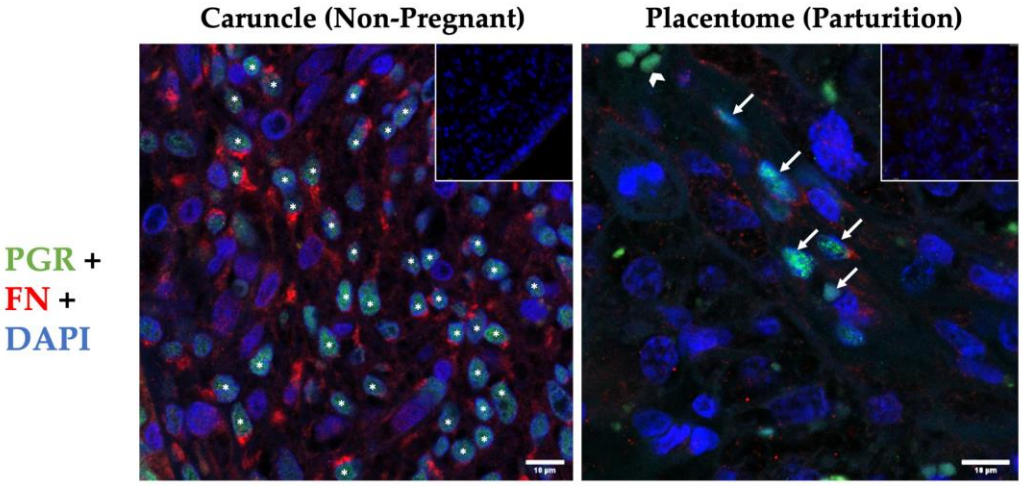

Figure S2. Three-color immunofluorescence staining of maternal stromal cells in caruncular and placentomal tissue sections, stained with DAPI and antibodies against PGR and FN. Tissue samples of NP caruncle (left figure) or P placentome (right figure) were stained with PGR (green) and mesenchymal marker VIM (red). Stromal myofibroblasts of the caruncle showed nuclear PGR staining (asterisks) and girdled cytoplasmic FN expression (red). In contrast, PGR-positive cells (arrows) presented weak cytoplasmic staining signal for FN in the placentomal stroma, compared to the caruncle section. Nuclei were counterstained with DAPI (blue). In merged images nuclear signals for PGR and DAPI overlap and appear in brighter white/turquoise color. Autofluroescenct signals were observed in erythrocytes of surrounding vessels (arrowhead). For each tissue section there was no background staining in the isotype control (insets). Scale bars = 10 µm.

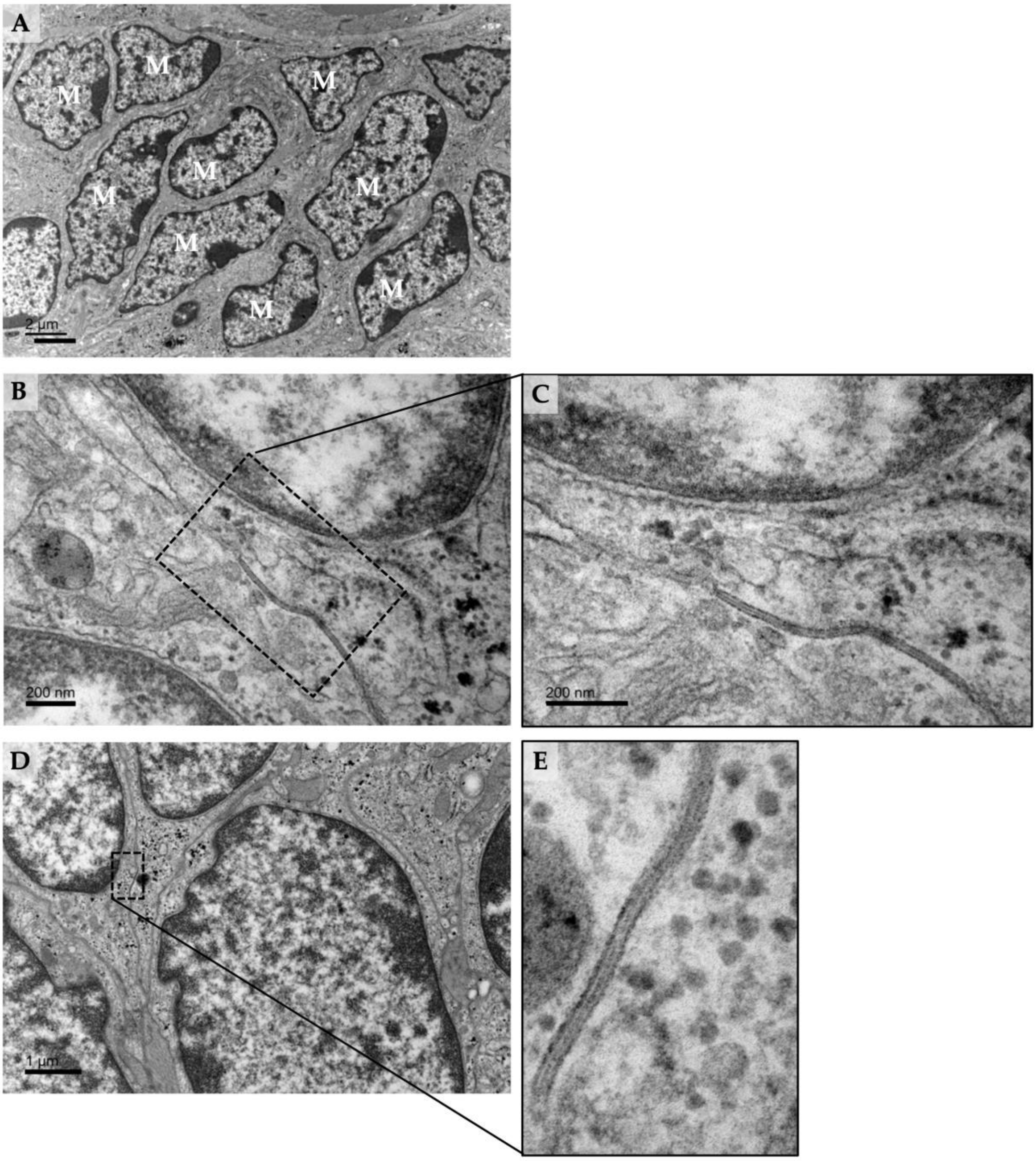

Figure S3. Transmission electron micrographs of bovine NP caruncle samples (A-E). **(A)** Ultrastructural overview of adjacent myofibroblasts (M). **(B)** Cell-Cell connection between two cells, showing a characteristic gap junction (doted rectangle) shown in a higher magnification. **(C)** Enlarged display of the gap junction, demonstrating representative features of hemichannels, represented as alternating bands to form the junction. **(D)** Area of adjacent myofibroblasts demonstrating the regions of cell-cell contacts. **(E)** Magnified extract of the cell connection, reflected as a gap junction with seven alternating bands (four dark and three bright bands).

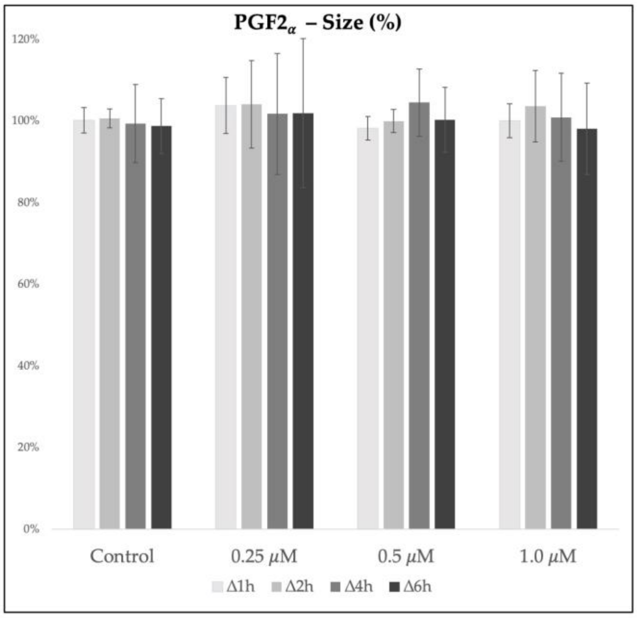

Figure S4. Arithmetic mean (± SD) area changes (in %) in PGF2_α stimulated, placentomal caruncle tissue slices through contraction assay experiments. Slices were analyzed after different incubation times (1h, 2h, 4h, 6h). Measurements started after preincubation and before the first treatment, which is considered as timepoint 0. There were no significant contraction effects in the PGF2_α -treated group at all stimulation timepoints ($p > 0.05$).

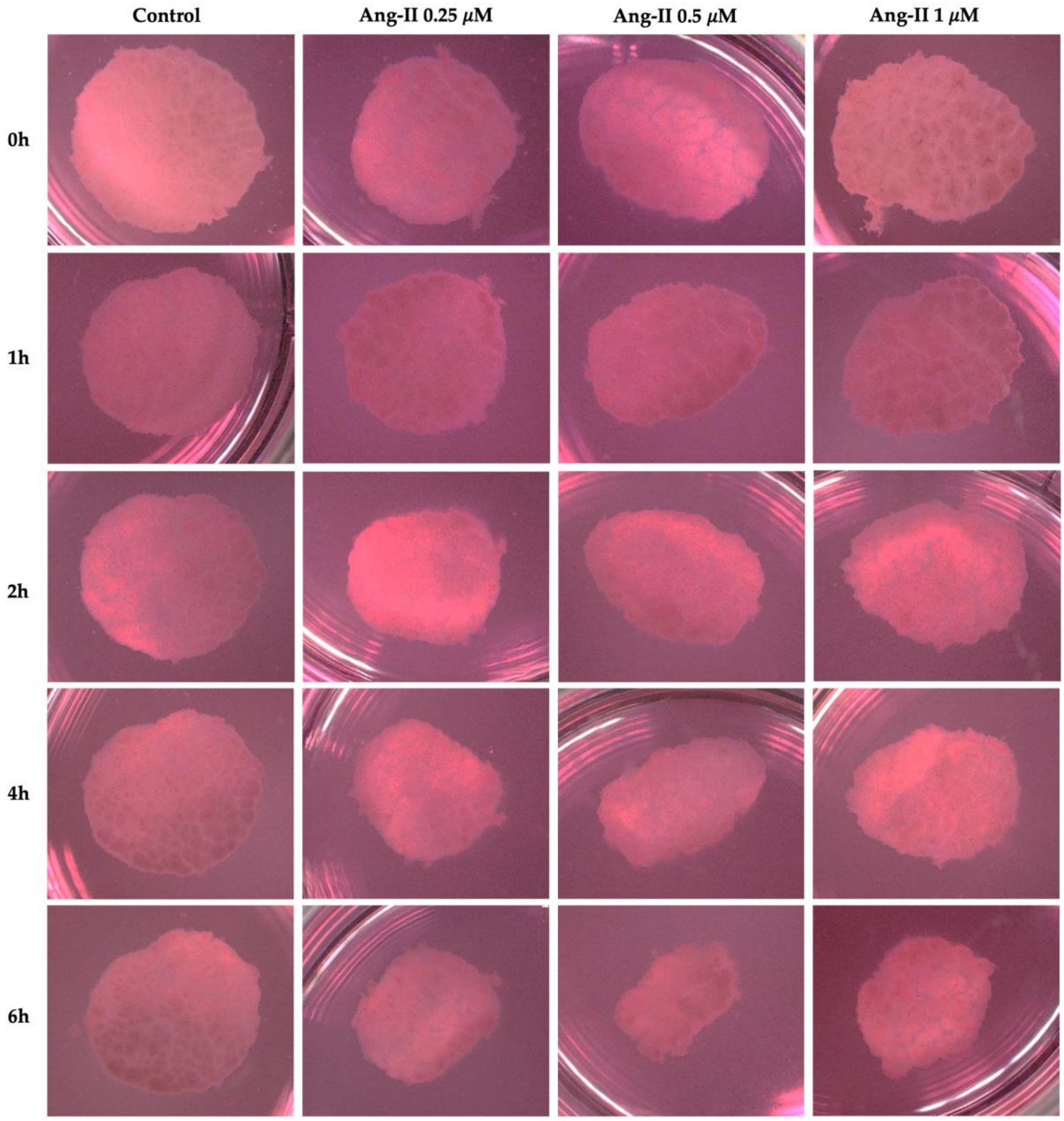

Figure S4. *In vitro* Ang—II treated caruncle tissue slices, obtained from placentomes, during time-course stimulation experiments. Controls were run simultanously, using a medium containing no adds. After preincuation period, first pictures were taken at time 0 (0h), prior to stimulation. To follow-up on tissue slice are variation (shrinkage) additional photographs were performed after 1h, 2h 4h or 6h after stimulation with Ang-II, to subsequently measure tissue areas (mm^2). As presented in the left row, no significant shrinkage was observed in the control slices during the incubation period of 6h ($p < 0.05$) . In contrast, Ang-II treatment initiated a time-dependent tissue shrinkage, seen via slice area variation in all treatment groups. Contraction activity increased steadily with progressive stimulation time. Thus, strongest contractile reaction manifested after 6h post stimulation with the three different Ang-II-concentrations, considered as very significant ($p < 0.01$). Slice diameter at time 0 = 8mm.

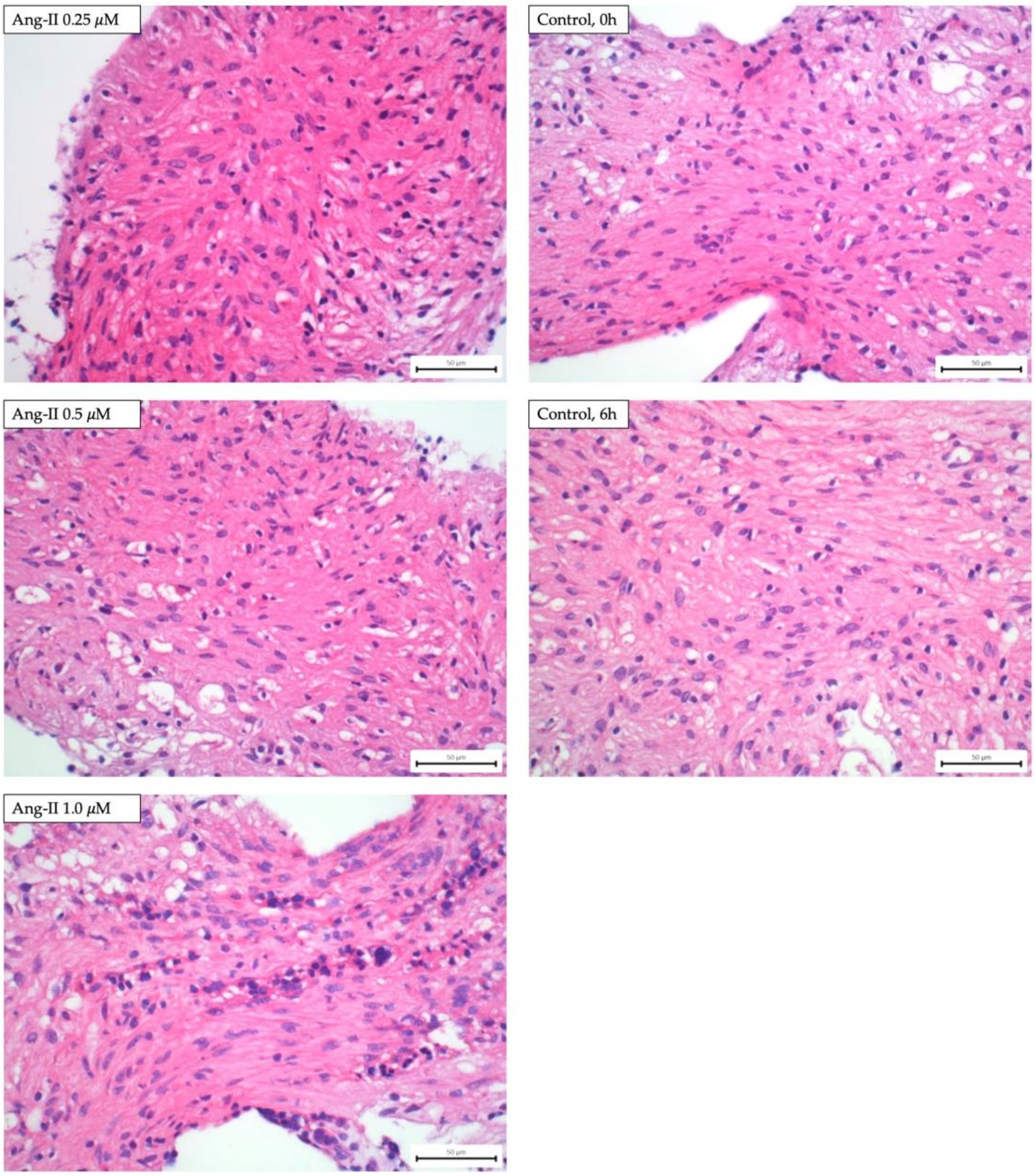

Figure S5. H&E-Staining of treated tissue slices for morphological evaluation. Processed and stained slices were analyzed after complete Ang-II incubation process (6h), i.e., fixation and staining was performed for the slices of 0.25 µM, 0.5 µM or 1 µM Ang-II treatment groups as well as for the control slice. Another control slice (collected at time 0) was additionally fixed for histological analysis. Scale bars = 50 µm.
